# Antioxidant and Synergistic Antidiabetic Activities of a Three-Plant Preparation Used in Cameroon Folk Medicine

**DOI:** 10.1155/2017/9501675

**Published:** 2017-04-26

**Authors:** Bruno Moukette Moukette, Vicky Jocelyne Ama Moor, Cacral Prosper Biapa Nya, Pauline Nanfack, Francine Tankeu Nzufo, Marcel Azabji Kenfack, Jeanne Yonkeu Ngogang, Constant Anatole Pieme

**Affiliations:** ^1^Laboratory of Biochemistry, Department of Physiological Sciences and Biochemistry, Faculty of Medicine and Biomedical Sciences, University of Yaoundé I, P.O. Box 1364, Yaoundé, Cameroon; ^2^Department of Biochemistry, Faculty of Sciences, University of Dschang, P.O. Box 96, Dschang, Cameroon

## Abstract

*Introduction.* Several plant preparations like a mixture of aqueous extracts of* Spilanthes africana; Portulaca oleracea*; and* Sida rhombifolia* are currently utilized in Foumban (West Cameroon) to manage diabetes. The aim of this study is to investigate the antidiabetic property of the aqueous mixture of three plant extracts (1 : 1 : 1) on streptozotocin induced diabetes rats.* Methods.* Diabetes was induced to rats by intraperitoneal (i.p.) injection of streptozotocin (STZ) at a dose of 50 mg/kg b.w. The diabetic rats received different dosages of the mixture of extracts for 21 days and glibenclamide 6.5 mg/kg b.w. as positive control.* Results.* The results showed that the mixture of extracts significantly (*p* < 0.05) decreased the level of the glycaemia, the total cholesterol, triglyceride, and LDL-cholesterol as well as MDA, AST, ALT, and creatinine levels. It also increased significantly the concentration of HDL-cholesterol, glutathione, and TAOS. A great reduction of the atherogenic indexes CT/HDL and LDL/HDL of the treated groups was observed. Each extract and the mixture demonstrated significant scavenging property on DPPH and OH radicals and present a good antioxidant property.* Conclusion.* The mixture of plant extracts has hypoglycemic, antioxidant, and hypolipidemic properties and can be used for the management of diabetes mellitus.

## 1. Introduction

Diabetes mellitus is a major illness with numerous clinical manifestations. It is one of the most common endocrine metabolic disorders, characterized by hyperglycemia due to defects in insulin secretion, action, or both [1]. According the International Diabetes Federation (IFD), the world diabetic population in 2015 was estimated to be 415 million and the prevalence increased from 4.7% in 1980 to 8.5% in 2015 [2]. The International Diabetes Federation (IDF) revealed that, in 2040, diabetes will affect 642 million people making it one of the leading causes of disability and death worldwide [2].

Several approaches of treatment of diabetes available include hormonotherapy (insulin) or by using glucose-lowering drugs such as alpha-glucosidase inhibitors, sulfonylureas, biguanides, and thiazolidinediones either in monotherapy or in combination [3]. However, the increase of side effects is one of the complications in the treatment of any systemic disorder. There is therefore an urgent need to search for new classes of compounds from phytomedicine without side effects for the disease [4]. Natural products are therefore a good alternative in contributing to solving the problem. There is a rich and different tradition in the use of herbal medicine for the treatment of several ailments in Cameroon. Several plant preparations were traditionally used to treat diabetes in Cameroon. Some of them were scientifically studied for hypoglycemic and antidiabetic activities [5, 6].

The concept of polyherbal formulation is well documented in the ancient literature. Traditional medicine systems generally assume that synergy effects of all ingredients of the plants will bring about maximum therapeutic efficacy [7]. Herbal combinations are more effective than the constituent herb used alone [8]. Compared to the single herb, the polyherbal formulation has better and extended therapeutic potential, enhances the therapeutic action, and reduces the concentrations of single herbs, thereby reducing adverse events [9, 10]. It is believed that the synergistic interactions between the constituents are responsible for the therapeutic efficacy [10].

In Cameroon, a plant mixture has been used in the west region of the country for many years as traditional medication for diabetes. This folk medicine is a mixture of three plant extracts:* Spilanthes africana (S. Africana)*,* Portulaca oleracea (P. oleracea),* and* Sida rhombifolia (S. rhombifolia)*.* Portulaca oleracea* (purslane) is widely distributed in several countries in Europe, Africa, and Asia where it is used as a potherb. It is an annual plant that produces flowers in the summer. In Cameroon, the stems and leaves of* P. oleracea* are consumed in several regions. It has been previously demonstrated that* Portulaca oleracea *contains flavonoids such as kaempferol, myricetin, luteolin, apigenin, quercetin, genistein, and genistin [11].* Sida rhombifolia* is a recurrent or sometimes yearly herb originating from the African and Asian tropic areas In India; potion of* S. rhombifolia*'s leaves has been demonstrated to have diuretic and aphrodisiac properties. The study led to the isolation of scopoletin, ethoxy-ferulate, kaempferol, kaempferol 11-methoxy-quindoline, quindoline, and the cryptolepine salt [11].* Spilanthes Africana *Murr. is well known as “toothache plant.” It has a long history of use as a folk remedy, for example, for toothache, rheumatism, fever, a snakebite, fracture, dysentery, and malaria remedy. In this genus, the major phytochemicals present are saturated and unsaturated alkyl ketones, alkamides, hydrocarbons, acetylenes, lactones, alkaloids, terpenoids, flavonoids, and coumarins [12].

Case report from local traditional clinics describes a betterment of the diabetes patients that were using this treatment. According to the information recorded from the healers, after collecting the tree plants, they are separately dried and crushed and then an equal quantity of each plant (two pinches) is taken to prepare the mixture in a glass of cool water. This information was used to design our study. According to the information recorded from the healers, after collecting the tree plant, they are separately dried and crushed and then an equal quantity of each plant (two pinches) is taken to prepare the mixture in a glass of cool water. This information was used to design our study. This medicinal preparation is widely consumed by the population without any scientific support on the effect on the medication on diabetes and without any data on the toxicity of the medicinal mixture. The plants:* P. oleracea*,* S. africana,* and* S. rhombifolia* have been individually investigated for their biological properties [11–13]. Hence, the present study aimed at investigating the antidiabetic properties of this folk medicine on streptozotocin diabetic rat models.

## 2. Materials and Methods

### 2.1. Plant Material

*Spilanthes africana *DC (Asteraceae),* Portulaca oleracea *Linn. (Portulacaceae), and* Sida rhombifolia *Linn. (Malvaceae) plants were collected in Foumban, west region of Cameroon. The plant samples were identified at the National Herbarium of Cameroon under the respective voucher numbers 33075/HNC, 17542/SRFcam, and 25373/SRFcam.

### 2.2. Extract Preparation

The leaves of each collected plant were dried at room temperature, pulverized, and sieved. The powders were then macerated separately in the distilled water in a ratio of 1 : 10 (W/V) for 48 h. The mixtures were filtered and concentrated in the oven at 55°C for until dryness. Each crude extract obtained was kept at 4°C. For the antidiabetic assay, the mixture solution of the three plant extracts (MTPE) was prepared in a ratio (1v : 1v : 1v). A serial dilution (20, 40, 80, 120, and 240 *μ*g/mL) of each extract and the mixture of the three plant extracts were used to determine the in vitro antioxidant activity.

### 2.3. Antidiabetic Activity of the MTPE

#### 2.3.1. Induction of Diabetes Mellitus

Diabetes was induced in vivo through intraperitoneal (i.p.) injection of streptozotocin (STZ) (Sigma Chemical Co., St. Louis, MO, USA) dissolved in 0.1 M of citrate buffer (pH 4.5) at a dose of 50 mg/kg b.w. The fasting blood glucose levels were determined by glycose oxidase peroxidase reactive trips (OneTouch). Control group of rats received the vehicle. Blood samples were collected by cutting the tip of the tail. The concentration of glucose was determined 3 days after induction and the rats which developed hyperglycemia (blood glucose higher than 2/5 g·L) were considered as diabetic and used in the experiment [14].

### 2.4. Treatment of Animals

Male albino Wistar rats weighing 200–250 g were used for the study. The animals were maintained at room temperature under the laboratory conditions and were fed with standard diet and the received water ad libitum. The animals were divided into 6 groups of 5 animals each—group I: untreated control (rats receiving distilled water); group 2: diabetic control (diabetic rats without treatment with extract but receiving distilled water); group 3: diabetic + glibenclamide (6.5 mg/kg b.w.* per os*.); groups 4–6: diabetic + MTPE at respective dose of 50 mg/kg 100 mg/kg and 200 mg/kg. The rats were treated with MTPE once per day for 21 days. Fasting blood glucose and body weight measurement were done during the study.

This study was carried out with approval from the animal Ethics Committee of University of Yaoundé I.

### 2.5. Biochemical Analysis

Fasting blood glucose level was determined on days 1, 7, 14 and 21 using OneTouch electronic glucometer (OneTouch). On day 21 after overnight fasting, the blood was collected after decapitation of rats under mild ether anesthesia. The serum was separated (centrifugation at 1500 ×g for 5 min) and used to determine the concentration of total cholesterol, triglycerides, and HDL-cholesterol using enzymatic colorimetric method (kits, Human Co., Germany). The serum level of LDL-cholesterol was calculated [15]. To investigate the effect of MTPE on liver and kidney function, the level of creatinine was determined with kits (ELITech Diagnostics, France) as well as the activities of aspartate transaminase (AST) and alanine aminotransferase (ALT) using enzymatic methods (kits, TECO diagnostic, USA). The in vivo antioxidant property of the MTPE was also carried out through the determination of the lipid peroxidation (MDA); reduced glutathione (GSH); and the total antioxidant status (TAOS) (Antioxidant Assay Kit, Sigma Co. (Catalog number CS0790).

### 2.6. In Vitro Scavenging and Antioxidant Activities

The in vitro scavenging activity of DPPH and OH radicals was determined using the standard methods followed by the potential antioxidant and phytochemical profile of the extracts [16–19].

### 2.7. Statistical Analysis

Data are expressed as mean ± SD. Data are expressed as mean ± SD. The biochemical parameters were analyzed statistically using SPSS software student's version 18 for Windows. The nonparametric test of Kruskal-Wallis and Dunnett's multiple comparison test were used to compared the different groups; *p* < 0.05 is considered statistically significant.

## 3. Results 

### 3.1. In Vitro Scavenging and Antioxidant Activities of* S. africana, P. oleracea, *and* S. rhombifolia* Extracts and MTPE

The results of the in vitro scavenging activity of the extract varied depending on the extract and the assay (Table 1). For the DPPH scavenging activity, the fifty percent inhibitory concentration (IC_50_) of the extract varied from 2.93 to 3.47 *μ*g/mL depending on the extract with* S. africana *demonstrating the lower concentration. Regarding the antioxidant activity, the MTPE demonstrated the highest antioxidant capacity for both FRAP activity (272.59 mEq BHT/g) and reducing power (Table 1). The phytochemical profile revealed the presence of phenolic compounds in the extracts such as flavonoids, tannins, and polyphenols and mucilage.

### 3.2. In Vivo Effects of MTPE on Diabetic and Normal Rats

#### 3.2.1. Effects of MTPE on Weight and Fasting Blood Glucose Levels of Diabetic Rats

**Figure 1:**
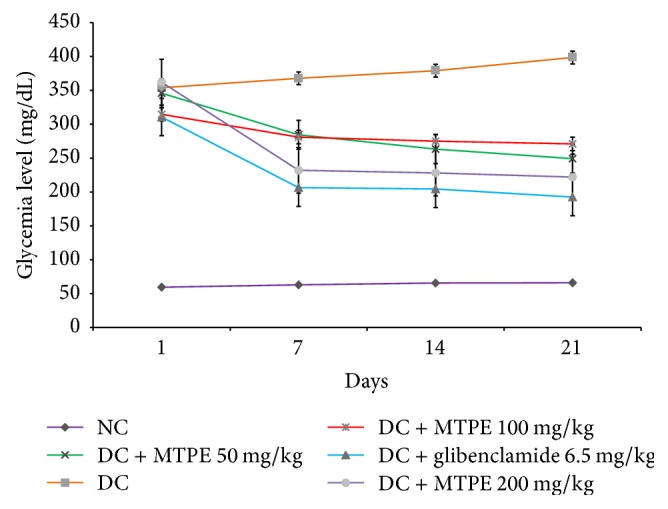
Effects of MTPE on blood glucose of normal and diabetic rats after 21 days of treatment. Values are expressed as means ± standard error (*n* = 5).

**Figure 2:**
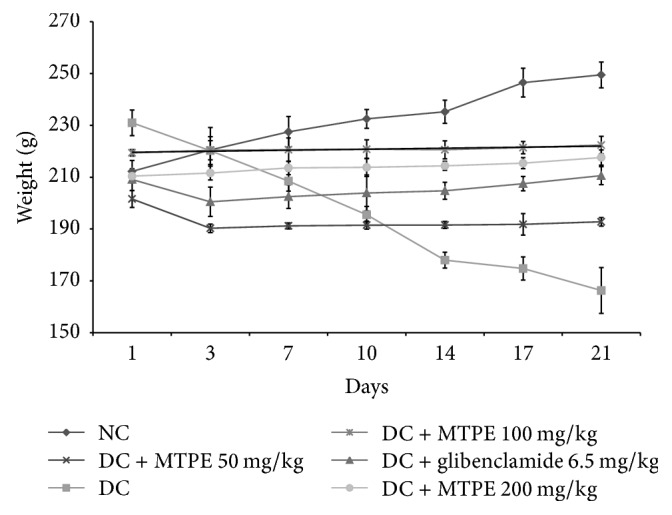
Effects of MTPE on weight variation of rats after 21 days of treatment. Values are expressed as means ± standard error (*n* = 5).

The effects of MTPE at different doses on fasting blood glucose levels of normal and diabetic rats are shown in Figure 1. The concentration of glucose of the untreated group of rats is lower and stable during the experiment compared to other groups. The oral administration of MTPE at the doses of 50, 100, and 200 mg/kg induced a significant decrease (*p* < 0.05) of fasting blood glucose level compared to control diabetic animals in which the glycaemia continues to increase. Moreover, a dose of 200 mg/kg MTPE demonstrated the significant and highest reduction of the level the blood glucose but this result remained lower compared to that of the group of rats treated with 6.5 mg/kg b.w. of glibenclamide. The administration of MTPE maintains or increases the body weight of diabetic rats during treatment compared to untreated diabetes rats in which loss of weight is significantly increased (Figure 2). Weight loss is a main sign of diabetes but its mechanism which is not elucidated may include loss of appetite, increased muscle waste, and loss of tissue proteins.

#### 3.2.2. Effects of MTPE on Lipid Profile of Normal and Diabetic Rats

The induction of diabetes caused a significant increase of triglycerides, total cholesterol, and LDL-cholesterol concentrations and a significant reduction of HDL-cholesterol level in the negative control group compared to others (Table 2). The administration of MTPE at dose of 50, 100, and 200 mg/kg significantly reversed the situation by decreasing the serum triglycerides, total cholesterol, and LDL-cholesterol levels and increasing the HDL-cholesterol. At 200 mg/kg the range reduction of triglycerides, total cholesterol, and LDL-cholesterol was between 1.68- and 11.81-fold that of negative control. The atherogenic index values were significantly decreased when the dose of administration of MTPE increases demonstrating the positive effect of the MTPE on lipid profile.

#### 3.2.3. Effects of MTPE on Markers of Hepatic Toxicity and Antioxidant Status of Normal and Diabetic Rats

The results of the effects of MTPE* on markers of hepatic toxicity and antioxidant status* on diabetic rats are shown in Table 3. The administration of glibenclamide and different doses of MTPE reduced significantly the concentration of the mentioned parameters. At 200 mg/kg of b.w. the enzymes activities of ALT and AST are lower compared to those of diabetic control and normal groups. The administration of MTPE or glibenclamide significantly decreased the levels of MDA in the serum while a significant (*p* < 0.05) increase of GSH and TAOS levels was observed. The lipid peroxidation index significantly decreases when the dose of MTPE given to rats increases (Table 3).

## 4. Discussion

Experimental diabetes induced by streptozotocin in rats is a model widely used to study various aspects of the disease. Diabetes mellitus is a metabolic disorder characterized by hyperglycemia and abnormal lipid and protein metabolism along with specific long-term complications affecting the retina, kidney, and nervous system [3]. Diabetes has been treated orally with several medicinal plants or their extracts based on knowledge from folk medicine for many years [20]. In this study, we investigated the hypoglycemic, hypolipidemia, and antioxidant properties of MTPE on experimental diabetes induced by streptozotocin. The oral administration of different doses of MTPE and glibenclamide to diabetic rats after 21 days decreased significantly the levels of blood glucose. The hypoglycemic activity found here could be attributed to the presence of active hypoglycemic agents found in the MTPE. Several studies demonstrated the hypoglycemic activity of each single plant extract of the mixture [21–23]. The phytochemical profile of MTPE present revealed the presence of flavonoids, polyphenols, tannins, mucilages, and other bioactive molecules whose hypoglycemic properties and stimulatory activity of insulin have been demonstrated [11]. Therefore, the hypoglycemia of MTPE could be attributed to the synergetic effect of each individual bioactive compounds present in each extract. The investigation of phytochemical composition of the extract of* S. africana, P. oleracea, *and* S. rhombifolia *demonstrated the presence of phenolic acids (ferulic acid and vanillic acid), coumarin (scopoletin), and triterpenoids (*β*-sitostenone and stigmasterol) [24, 25]; flavonoids (quercetin, luteolin); alkaloids; organic acids (catechol, caffeic acid) and vitamins and minerals [24–26] and amino acids and fatty acids (linolenic acid, linoleic acid, and palmitic acid) [26]. Studies showed that some of these molecules have antioxidant and protective effects against DNA and protein damage [27]. The presence of these bioactive molecules in MTPE may operate either through the cryoprotective effects on the pancreatic beta cells or through the combination of their abilities to stimulate the secretion of insulin or protect cellular functions [27]. Diabetes is associated with profound alterations in the plasma lipid and lipoprotein profile and increased risk of coronary heart disease [28]. Hypercholesterolemia and hypertriglyceridaemia have been reported to occur in streptozotocin diabetic rats [29]. Our study showed that MTPE could reverse the hyperlipidemia and thus leads to a decrease of the risk of complications of diabetes [30]. The improvement of lipid profile might be directly or indirectly related to the reduction of blood glucose levels. Lowering of serum lipid levels through dietary or drug therapy seems to be associated with a decrease in the risk of vascular disease and related complications [30]. The increase of the atherogenic index is linked to an increase in oxidative damage [31]. Rats treated with MTPE and glibenclamide showed an increase of HDL-cholesterol and a reduction of atherogenic index and LDL-cholesterol and triglycerides and total cholesterol. The hypocholesterolemia properties of* S. africana, P. oleracea, *and* S. rhombifolia* extracts have been reported [22, 32, 33]. Also, study showed that mucilage present in MTPE can bind bile acids, prevents their absorption, and reduces the blood cholesterol level. The hypoglycemia effect of the MTPE could be attributed to its ability to restore the function of pancreatic tissues by increasing insulin output, inhibiting the intestinal absorption of glucose, or enhancing metabolism of insulin-dependent processes. The serum creatinine and transaminases (ALT and AST) are used as good indicators of the renal and hepatic functions, respectively. The induction of diabetes with streptozotocin causes the lysis hepatocytes cells which release transaminases indicating impaired liver function which may cause hepatic damage [34]. Also, diabetic complications, such as increased gluconeogenesis and ketogenesis, may be due to elevated transaminase activities [35]. The oral administration of the MTPE or glibenclamide significantly decreased ALT and AST activities and restored all these parameters towards normal levels in a dose-dependent manner as well as creatinine a marker of the kidney function [35]. Oxidative stress in diabetes mellitus has been shown to coexist with a reduction in the endogenous antioxidant status and this is caused by dysfunction of pancreatic beta cells [36]. It plays an important role in the pathogenesis of diabetes complications such as atherosclerosis, nephrotic, neurological, and retinal disorders [34]. Lipid peroxide-mediated tissue damage has been observed in the development of both type I and II diabetes mellitus. The MDA is the biomarker of lipid peroxidation most used. The MDA levels of untreated diabetic group of rats were higher than those of the control demonstrating an increase of lipid peroxidation and free radical productions [37–39]. With the administration of MTPE a significant decrease of the concentration of MDA has been noted while those of glutathione and TAOS are increased significantly in the concentration dependent manner. The reduction of MDA level was higher which could be attributed to several phytochemical bioactive compounds such as polyphenol and flavonoids which scavenge the free radicals production during lipid peroxidation. Other antioxidant mechanisms of these bioactive molecules demonstrated in vitro include the ability to provide the H^+^ ion or an electron to the oxidant molecule [40]. It has been reported that the phenolic compounds could prevent oxidative stress produced by streptozotocin to induce death of beta cell, by increasing their viability through protection of DNA or by enhancing the natural antioxidant system [41]. GSH plays an important role in the endogenous nonenzymatic antioxidant system which acts primarily as a reducing agent and detoxifies hydrogen peroxide in the presence of an enzyme, glutathione peroxidase. The increase of GHS level after administration of MTPE implies either the increase of its endogenous synthesis or increase of antioxidant capacity through the implication of natural antioxidant such as polyphenols and flavonoids. Polyphenolic compounds are a large group of phytochemicals found in a variety of plants, vegetables, and fruits. It was reported that flavonoids and polyphenolic compounds can suppress insulin resistance, possibly mediated via activation of PPAR*γ*, reduced oxidative stress, and enhanced glucose uptake and insulin sensitivity [42]. The hypoglycemic action can be due to release of insulin, insulin-sensitizing action, a combination of both, or increase of the antioxidant capacity of plasma by strengthening the potential of antioxidant enzymes. Further chronic studies need to be undertaken to determine the mechanism of action of MTPE.

## 5. Conclusions 

In conclusion, the aqueous MTPE exhibited significant hypoglycemic antioxidant and hypolipidemic properties. This mixture has not showed the increase of some liver enzymes in vivo. However further experiments are required to identify the active molecules and elucidate the effect of extract on insulin.

## Figures and Tables

**Table 1:** Information of plants used: antioxidant and antiradical properties.

Plant species/family/ (voucher specimen)^a^	Vernacular names/area in Cameroon/place of collection and date	Part of plant used (extraction yield in %)^b^	In vitro antiradical and antioxidant activities of the extract			
			IC_50_ (*μ*g/mL)		Antioxidant (at 50 *μ*g/mL of extract)	
			DPPH	OH	Reducing power (700 nm)	Antioxidant potential (mgEq/BHTg of extract)
*S. africana* DC (Asteraceae) 33075/HNC	*“Mba kouom”* (Bamoun), Foumban; November 2010	Leaves 20.68	2.93	3.47	0.15	125
*P.oleracea * Linn. (Portulacaceae) 17542/SRFcam	*“Kepingoup, Koupugoup”* (Bamoun), Foumban; November 2010	Leaves 13.29	3.07	3.24	0.14	127.5
*S. rhombifolia * Linn. Malvaceae 25373/SRFcam	*“Sesam, Cisen”* (Bamoun), Foumban; November 2010	Leaves 13.36	2.79	3.21	0.17	132
*Mixture (1 : 1 : 1)*			2.97	5.62	0.12	135
*Vitamin C*			1.65	2.9	0.24	220

^a^Plants were identified at the Cameroon National Herbarium (HNC).

^b^The percentage of the aqueous extract.

**Table 2:** Effects of MTPE on lipid profile of normal and diabetic rats.

Groups	Parameters				TC/HDLC	LDLC/HDLC
	HDL-cholesterol (mg/dL)	LDL-cholesterol (mg/dL)	Triglycerides (mg/dL)	Total cholesterol (mg/dL)		
Untreated control	43.20 ± 2.16	17.00 ± 2.12	41.00 ± 1.58	69.20 ± 1.92	1.43 ± 0.70	0.25 ± 0.58
Diabetic control	11.80 ± 1.30^*∗*^	78.20 ± 5.89^*∗*^	134.80 ± 3.63^*∗*^	117.60 ± 5.02^*∗*^	7.74 ± 1.24^*∗*^	4.91 ± 1.03^*∗*^
Diabetic + glibenclamide 6.5 mg/kg	40.20 ± 1.78^a^	30.40 ± 2.79^a^	56.80 ± 3.83^a^	82.80 ± 2.16^a^	1.93 ± 0.88^a^	0.64 ± 0.90^a^
Diabetic + MTPE 50 mg/kg	34.00 ± 3.08^a^	30.60 ± 4.92^a^	119.40 ± 7.50^a^	89.40 ± 4.21^a^	2.83 ± 2.26^a^	1.01 ± 0.17^a^
Diabetic + MTPE 100 mg/kg	41.20 ± 1.78^a^	11.80 ± 2.04^a^	86.60 ± 1.81^a^	71.00 ± 2.23^a^	2.16 ± 0.22^a^	0.65 ± 0.55^a^
Diabetic + MTPE 200 mg/kg	48.20 ± 3.03^a^	6.60 ± 5.47^a^	47.20 ± 1.78^a^	65.00 ± 3.00^a^	1.53 ± 0.07^a^	0.31 ± 0.17^a^

Values are expressed as means ± standard error (*n* = 5).

Values affected with letter a are significantly different (*p* < 0.05) from the diabetic control.

Values affected with *∗* are significantly different (*p* < 0.001) from the normal control.

**Table 3:** Effects of MTPE on markers of toxicity and antioxidant activity after 21 days of treatment.

Groups	Parameters						
	ALT (UI/L)	AST (UI/L)	Creatinine (mg/L)	MDA (*μ*M)	Glutathione (*μ*M)	TAOS (*μ*M)	Total protein (g/L)
Untreated control	64.20 ± 2.48	78.80 ± 6.37	22.0 ± 0.44	1.60 ± 0.25	9.28 ± 1.96	23.00 ± 0.02	97.80 ± 4.32
Diabetic control	156.20 ± 2.68^*∗*^	205.20 ± 0.44^*∗*^	130.0 ± 0.70^*∗*^	4.48 ± 0.63^*∗*^	5.10 ± 0.70^*∗*^	7.00 ± 0.04^*∗*^	40.80 ± 2.16^*∗*^
Diabetic + glibenclamide 6.5 mg/kg	68.20 ± 3.19^b^	87.40 ± 4.33^b^	44.0 ± 0.54^b^	2.94 ± 0.28^a^	14.22 ± 3.47^a^	3.90 ± 0.03^a^	80.20 ± 2.16^a^
Diabetic + MTPE 50 mg/kg	117.00 ± 8.94^b^	84.00 ± 5.78^b^	68.0 ± 0.44^b^	3.46 ± 0.42^a^	9.06 ± 1.03^a^	16.00 ± 0.03^a^	75.60 ± 4.27^a^
Diabetic + MTPE 100 mg/kg	95.00 ± 2.82^b^	84.80 ± 2.94^b^	50.0 ± 1.22^b^	2.34 ± 0.41^a^	13.90 ± 2.35^a^	23.00 ± 0.02^a^	81.00 ± 4.24^a^
Diabetic + MTPE 200 mg/kg	60.00 ± 4.18^b^	59.20 ± 3.03^b^	38.0 ± 0.44^b^	1.72 ± 0.17^a^	15.50 ± 2.41^a^	26.00 ± 0.01^a^	90.40 ± 3.28^a^

Values are expressed as means ± standard error (*n* = 5).

Values affected with the letters a and b are significantly different (*p* < 0.005) from the diabetic control.

Values affected with *∗* are significantly different (*p* < 0.001) from the normal control.
